# Ten facts from critical and interpretive social sciences for environmental research

**DOI:** 10.1016/j.isci.2025.112736

**Published:** 2025-05-23

**Authors:** Jasper Montana, E.A. Welden, Lea May Anderson, Aoife Bennett, Andrea Byfuglien, Sophie Bhalla, Hannah Fair, Beth Greenhough, Caitlin Hafferty, Mark Hirons, Eric Mensah Kumeh, Victoria Maguire-Rajpaul, Constance L. McDermott, Mari Mulyani, Laura Picot

**Affiliations:** 1 Australian National Centre for the Public Awareness of Science, Australian National University, Acton, ACT 2612, Australia; 2 School of Geography and the Environment, University of Oxford, Oxford, Oxfordshire OX1 3QY, UK; 3 Department of Geography, University of California-Berkeley, Berkeley, CA 94720-4740, USA; 4 Leverhulme Centre for Nature Recovery, University of Oxford, Oxford, Oxfordshire OX1 3QY, UK; 5 Environmental Change Institute, University of Oxford, Oxford, Oxfordshire OX1 3QY, UK; 6 Amazon Sustainability Research Institute, Universidad de Ingeniería & Tecnología, Lima 15063, Peru; 7 Morwick G360 Groundwater Research Institute, University of Guelph, Guelph, ON N1G 2W1, Canada; 8 School of Geography and Environmental Science, University of Southampton, University Road, Southampton SO17 1BJ, UK; 9 Keble College, University of Oxford, Oxford, Oxfordshire OX1 3PG, UK; 10 Global Sustainability Institute, Anglia Ruskin University, Cambridge, Cambridgeshire CB1 1PT, UK

**Keywords:** Environmental science, Social sciences

## Abstract

The social sciences are crucial contributors to environmental research. Collectively, they provide insights on the economic, cultural, political, and psychological dimensions of sustainability challenges. Yet, efforts to mainstream the social sciences in environmental research are missing the diversity of social science scholarship. Here, we contend that the critical and interpretive social sciences —which question and rethink established paradigms and power structures— have an invaluable, yet still underutilized, role. We propose that rethinking the focus, conduct, and goals of environmental research recognizing 10 facts from the critical and interpretive social sciences can help environmental research to better support desired transformative change for the benefit of both people and planet.

## Graphical abstract

**Figure:**
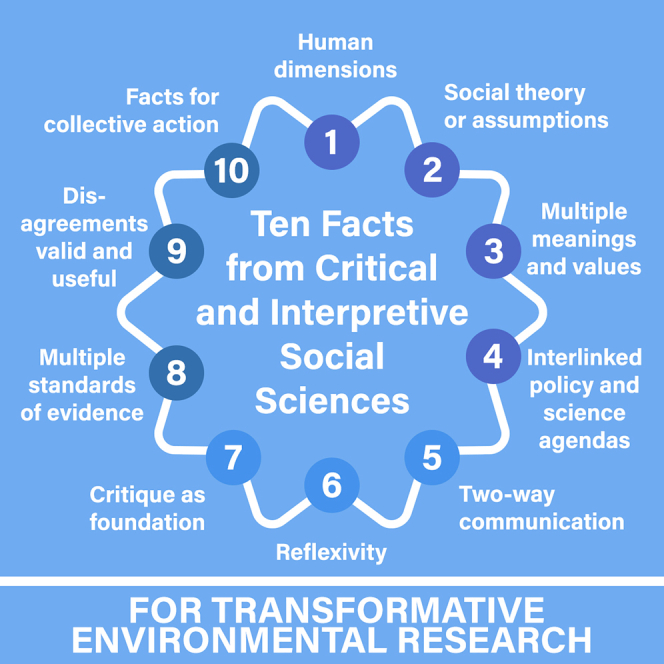

## Introduction

Sustainability challenges such as climate change, pollution, and biodiversity loss encompass an array of urgent and interlinked environmental, economic, and social problems.^1^^,^^2^^,^^3^^,^^4^ Numerous global science initiatives have now concluded that the threat posed by these challenges means business-as-usual is untenable. They contend that sustaining human wellbeing for the long term will require the fundamental reorganization of social, technological, and economic systems – or what has been termed “transformative change”.^5^^,^^6^^,^^7^ However, the capacity for environmental research as it is currently configured to enable transformative change at scale and haste remains uncertain.^8^^,^^9^^,^^10^

**Figure 1:**
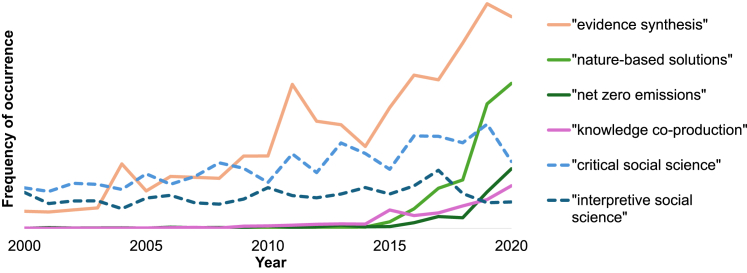
Google n-gram showing frequency of key terms in English-language publications between the years 2000 and 2020 related to reforms in environmental research and education, including: a shift to solutions represented by the terms “net zero emissions” and “nature-based solutions”; the promotion of ideals around equity, diversity and inclusion reflected in the term “knowledge co-production”; and the push for policy impact represented by the term “evidence synthesis” “Critical social science” and “interpretive social science” (dashed lines) are included for illustrative comparison.

Environmental research has rapidly evolved in recent years. Since at least the turn of the millennium, the recognized urgency of environmental challenges has prompted concerted efforts to enhance the usefulness and usability of research.^11^^,^^12^^,^^13^ Major developments have included reforms to the focus, conduct, and goals of environmental research, including: a growing focus on solutions to environmental problems; a recognition of the importance of equity, diversity, and inclusion in research; and an emphasis on evidence synthesis as a pathway to policy impact. Arguably, all three reforms have experienced a marked inflection in the decade since 2015 (Figure 1).

Efforts to improve the usefulness and usability of environmental knowledge have been accompanied by a greater recognition of the need to include the social sciences in environmental research and education.^14^^,^^15^^,^^16^^,^^17^^,^^18^^,^^19^^,^^20^^,^^21^^,^^22^^,^^23^ The pursuit of solutions has been exemplified in the 2015 reorientation of the Intergovernmental Panel on Climate Change (IPCC),^24^ as well as the proliferation of research into net zero emissions^25^ and nature-based solutions^26^. In each of these cases, the social sciences have been recognized as a necessary dimension, such as for understanding the enablers and barriers of the uptake of solutions.^27^ The leadership of the IPCC, for example, has made it clear that climate change adaptation and mitigation options “encompass not just technological but [also] social and institutional questions and economic considerations”.^28^^: 107^ Likewise, the importance of equity, diversity, and inclusion is exemplified by the wide-spread adoption of knowledge co-production approaches^29^ and echoed in commitments to inclusion of diverse perspectives in organizations such as the Intergovernmental Science-Policy Platform on Biodiversity and Ecosystem Services (IPBES).^30^ There have been significant insights drawn from the social sciences on principles and practices to guide these reforms.^29^^,^^31^ Finally, the pursuit of policy impact through research synthesis^32^ has also sought to incorporate research from the social sciences to provide a more holistic worldview offered by synthesis processes, particularly in some global environmental assessments.^16^^,^^33^^,^^34^^,^^35^^,^^36^

However, the extent to which existing reforms to environmental knowledge production offer sufficient opportunities for mainstreaming the full breadth of the social sciences remains an open question.^9^^,^^37^^,^^38^^,^^39^^,^^40^ The social sciences are not a monolithic entity that is able to be represented by a single researcher (or even a handful of researchers), nor is it characterized by a single way of doing research.^41^ Instead, the social sciences are inherently plural, encompassing a wide array of theories, philosophies and methodologies.^41^ In considering progress toward the mainstreaming of the social sciences into environmental research, there is a need to evaluate whether the diversity of the social sciences themselves has been adequately taken into account and ensure that there is tailored support for accommodating social science scholarship in all its forms.^18^

Here, we draw particular attention to the critical and interpretive social sciences —a heterogeneous and multi-sited approach to scholarship that questions and rethinks established paradigms and power structures— as a valuable resource for enabling desired transformative change. Specifically, we reflect on progress in mainstreaming the social sciences and examine whether recent reforms to enhance the societal impact of environmental research provide appropriate conditions for their inclusion. We contend that recent adjustments to the focus, conduct, and goals of environmental research could be revised to better enable contributions from the critical and interpretive social sciences. To support this outcome, we frame our proposition as a series of “facts” from the critical and interpretive social sciences that may help to raise awareness of the different worldviews that social scientists bring to the research process, provide the conditions for them to fruitfully contribute, and more broadly enhance a culture of communication across disciplines to enable desired transformative change.

## Diversity is a strength of the social sciences

The social sciences span a wide range of disciplines including, but not limited to, anthropology, business studies, cultural studies, economics, human geography, political science, psychology, and sociology.^18^ The social sciences are unified by their interest in the social world. However, how social scientists define and approach this social world differs greatly. Social scientists are diverse in what they consider to be relevant social actors for study: some social scientists see their work as focusing mostly on humans and their institutions, for example, research on human poverty or electoral politics; other social scientists may center their work on non-human actors, including physical and imagined entities that shape and are shaped by humans and their societies. This could include, for example, research focused on animals^42^ or energy systems^43^ as participants in the social world. As such, the social sciences are not necessarily defined by the ‘social objects’ that capture their scholarly attention.^44^^,^^45^ This is particularly pertinent for environmental research, where social scientists often take non-human actors as their starting point.^46^ Here, we refer to the social sciences as a shorthand to describe fields of research that locate their enquiry in the social world, meaning that human organizations, perceptions, values, and interests are seen to matter alongside physical processes.

**Figure 2:**
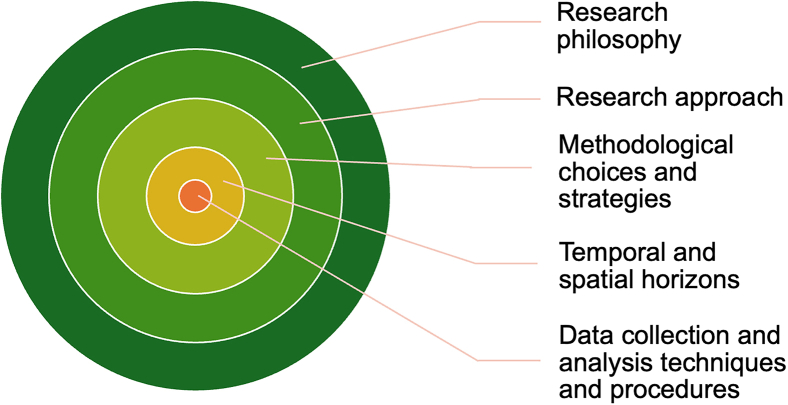
The research onion Adapted from Saunders et al.^47^

Like other domains of research, the social sciences maintain great diversity in their use of methods, frameworks, theories, and scales.^41^ Where the social sciences stand out most is around the diversity of research philosophies that they embrace.^41^ A research philosophy refers to the “system of beliefs and assumptions about the development of knowledge” that is adopted by a researcher.^47^^: 131^ Research philosophies —or the world view within which research is carried out— can be understood through the heuristic of a “research onion” that shows the nested layers of explicit and implicit choices made in carrying out research (Figure 2). According to this framework, the chosen research philosophy surrounds all other choices about what to study, where, and how.^47^ Research philosophies encompass assumptions that are made in research about the reality of the world under investigation (ontological assumptions) and the means by which knowledge can be meaningfully made about that reality (epistemological assumptions).^47^ Research philosophies are then operationalized through lower-order questions about the research approach (i.e., inductive or deductive research), the methodologies (i.e., mixed-method, qualitative or quantitative research), and the specifics of how, where, and when research will be carried out.

Social science disciplines can actively deploy a range of different research philosophies. The field of business studies, for example, commonly teaches and applies at least five different research philosophies, including “positivism”, “critical realism”, “interpretivism”, “postmodernism”, and “pragmatism”.^47^ Here, a positivist research philosophy is premised on the assumed existence of a universal external reality that is observable and measurable through the scientific method.^47^ This positivist approach is also considered to be the dominant research philosophy of the natural sciences.^41^ Other noted research philosophies (also sometimes termed paradigms) diverge from this assumed universal reality, and to varying degrees take (sometimes multiple) realities as perceived through theoretical lenses and cultural filters, which require interpretation, interrogation, and explanation. In this article, we focus on some of these subdomains of social science research that do not follow a strictly positivist research philosophy, which we refer to as the critical and interpretive social sciences.

The critical and interpretive social sciences are found within a range of social science disciplines and are shaped by the cross-pollinations of theory, ideas, and approaches within the social sciences and humanities.^48^ In a recent article, Turnhout (2024) defined the critical social sciences and humanities as “forms of research and scholarship that draw from alternative relational, critical, poststructuralist, and constructivist paradigms, including feminism, posthumanism, decolonial and Indigenous scholarship, science and technology studies, and political ecology”^49^^: 4^. We develop a similar definition here, recognizing that the critical and interpretive social sciences combine an analytical impetus to unpack and explain power differentials and inequities brought about by specific social conditions or interventions.^15^ This includes an understanding of how language, culture, and history profoundly influence the ways individuals and groups perceive, and live within, their realities.^50^ Many areas of the critical and interpretive social sciences are closely tied to (if not inseparable from) scholarship in the humanities.^21^^,^^49^ Although the humanities are not explicitly discussed in detail here, they remain a necessary partner of critical and interpretive social sciences, and of environmental research more broadly.^51^^,^^52^

The critical and interpretive social sciences are particularly well equipped to enable the kinds of transformative change deemed necessary to respond to current sustainability challenges.^6^^,^^15^^,^^20^^,^^22^^,^^50^^,^^53^ Critical and interpretive social sciences are unified by their engagement with theory about the workings of the social world^54^ and a philosophy of critical reflection that is seen as a precursor to emancipatory change^55^; they provide resources that align with foundational work on leverage points (or places to intervene in a system to bring about change) that recognize paradigm shifts and the distribution of power as significant loci of transformations.^56^ As such, they can deploy important revisionist thinking to enable desired transformative change^6^^,^^15^^,^^20^^,^^22^^,^^50^^,^^53^ through their theoretical focus on key social variables such as politics, power, culture and human values. Scholarship from the critical and interpretive social sciences, for example, can show how our understanding of a given problem (such as climate change) is culturally shaped through media narratives and provide the theoretical resources to make sense of these effects (Box 1). Through their capacity to explore the operation of politics, power, culture, and human values, the critical and interpretive social sciences also have potential to help identify and evaluate new ways of doing environmental research.^9^^,^^40^

Box 1 **Two research philosophies in climate change communication research**

The contrast between the positivist and critical social sciences is illustrated in two recent articles in *One Earth* focused on climate change communication. In the first, the authors use a survey to identify individual’s perceived urgency of climate change and the link with their support for different climate change mitigation policies.^57^ In the second, the authors examine the underlying meaning in climate change messaging advanced through public communications by a fossil fuel company.^58^ In the first example, the researchers adopt a positivist philosophy operationalized through a “statistical approach [and] experimental procedures” to analyze quantitative data and derive facts about individual perceptions. In the second example, the authors adopt an interpretive philosophy operationalized through an “inductive, qualitative approach” to explore the meaning that is made within qualitative data represented by climate change communications of a company.

## Social science representation is constrained in environmental research

**Figure 3:**
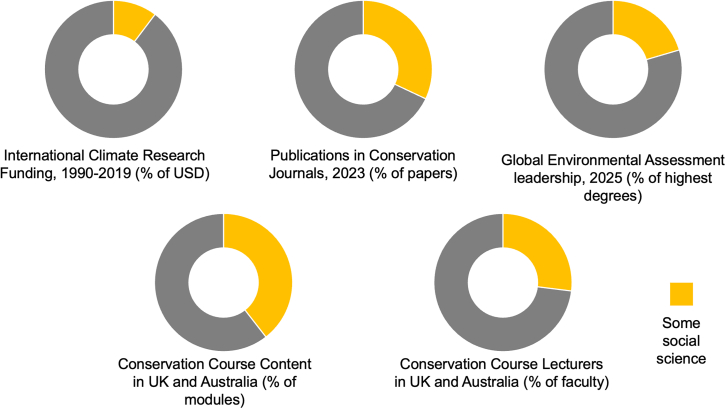
Compilation of graphs showing representation of the social sciences in environmental research Of international climate change funding between 1990 and 2019, 4.6 billion USD was directed to social sciences compared to 40 billion USD for the biophysical sciences. Of journal articles published in leading conservation journals in 2023, 32% included some social science. Of the 35-strong academic leadership of the IPCC and IPBES, only 9 had a higher degree that included some social science and none had a purely social science higher degree. Of university-based conservation courses in the UK and Australia, 60% of courses included some social science content compared to 92% for natural science content. And for the social science subject areas, only 31% were taught by faculty with social science backgrounds, compared to 84% of biological science courses and their faculty. Data sources set out in supplemental information.

Despite recognition of the importance of the social sciences, evidence suggests that they are still poorly represented in environmental research – particularly compared to the biophysical sciences, including disciplines such as biology, chemistry, earth sciences or physics. Research shows that the social sciences continue to receive significantly less funding,^59^ are published less frequently in environmental journals,^60^^,^^61^^,^^62^ are not well represented in the leadership of global environmental assessments,^63^ and are given less coverage and by lesser qualified lecturers in university-based education^37^^,^^64^ (Figure 3). Conversely, within the social sciences, the publication of environmental research in top journals is also still an outlier rather than a norm.^65^ However, fields such as sociology^66^ and geography^67^ have developed new journals to increase the visibility of environmental research as part of these core disciplines.

These comparative analyses do not disaggregate the critical and interpretive social sciences from other social sciences with positivist research philosophies. However, there is some indication that the critical and interpretive social sciences are even more marginalized. For example, an analysis of over 100,000 articles from the environmental social sciences between 1990 and 2022 found that significantly fewer articles “challenge the status quo or ask more abstract, theoretical questions” compared to those that focus on “changing particular governance and/or market mechanisms to produce better environmental outcomes”.^68^ This finding is supported by an analysis of the academic literature on socioecological systems between 2010 and 2015, which found that across the papers analyzed, key social variables of the critical and interpretive social sciences (i.e., culture, politics, and power) were significantly underrepresented compared to other social science concepts such as demographics, resource use, economics, and behavior.^69^ In global environmental assessments, there is similar evidence of marginalization. Research in 2011 found a clear bias in the IPCC’s third assessment report toward economic research, rather than other social sciences.^70^ However, there is some indication that the overall proportion of general social science has more recently increased in the IPCC’s Working Group 1 reports (i.e., from 0.47% to 1.61% of cited material between the fifth and the sixth assessment cycles).^71^ Nevertheless, a more recent account of conditions within IPCC author groups from 2024 suggests that cultural attitudes toward “newcomers” in the IPCC, including those from the social sciences, meant that these experts “remained at the margin of the conversations […] around commonly held assumptions and established practices”^72^^: 1013^. These resulting imbalances in the distribution of power and resources are considered an “injustice” that prevents some researchers from being recognized, represented, and fully able to participate in environmental research.^73^

There is evidence that current solutions to developing more useful and usable environmental research that ostensibly foreground opportunities for the social sciences to be included may be reinforcing the limited representation and influence of some parts of the social sciences.^9^^,^^30^^,^^40^ Existing reforms to the focus, conduct, and goals of environmental research may paradoxically create more rigidity in the research system, creating fewer opportunities for transformative change rather than more.

First, scholars have noted that framing research around solutions has the effect of “closing down” discussions about the nature of environmental problems, making them blind to alternative solutions and perspectives, especially more transformative agendas.^74^ In climate research, an emphasis on solutions has been blamed for knowledge being increasingly expressed in “simplified and narrow ways that privileges predictive natural sciences over interpretative qualitative social sciences and humanities”.^27^ An analysis of recent environmental social science scholarship has likewise found a growth in research on policy-relevant topics which favor incremental environmental improvements that align with existing political and economic orders, rather than transformative agendas.^68^ Other scholars have drawn particular attention to the way that many environmental issues are framed through the pursuit of physical targets, such as net zero emissions and zero deforestation, which are seen to limit the potential for the critical and interpretive social sciences to introduce sociocultural understandings of sustainability challenges that could widen the conversation beyond command and control policies and technical solutions.^75^ The capacity for research in the critical social sciences to think more expansively about human-nature relations, including breaking down dichotomies between the natural and the social, can enable the re-thinking of environmental problems in important ways.^76^^,^^77^^,^^78^

Second, despite decades of academic work into research systems, participatory methods, and the navigation of power inequalities, in practice, there are well known perils associated with agendas for greater inclusion. Scholars have shown that in many cases inclusion can be tokenistic for marginalized voices, lead to the co-opting of others’ agendas, focus less on listening to other perspectives and more on convincing others to see things the dominant way, or exploiting the authority of marginalized groups without ensuring their agency within the process.^79^^,^^80^^,^^81^ As a recent analysis of transnational conservation non-governmental organizations found, social scientists are often working within a framework of “asymmetrical interdisciplinarity”, where they are required to “engage in extra hidden labor as they seek to disrupt hegemonic ways of conceptualizing and practicing conservation”.^82^^: 268^ Furthermore, inclusion approaches that depend on a numerical approach to achieving disciplinary balance —as can be the case in global environmental assessments^63^— have been found to obscure the need to also provide resources and decision-making power for social science participants to define their contributions and establish what has been termed “epistemic belonging” within these knowledge systems.^83^

Third, scholars have noted that while evidence synthesis ostensibly presents opportunities for enhancing the inclusion of the social sciences, these opportunities can be fundamentally undermined by an overemphasis on consensus-based knowledge production.^30^^,^^84^ A focus on consensus building leads to potentially vital knowledge and insights being missed because they do not fit the dominant evidence framework. While scholars adopting positivist research philosophies might see individual research projects as producing a new puzzle piece that can be put together to form a larger puzzle, those from more critical and interpretive traditions are more likely to see themselves as working on distinct and contradictory puzzles altogether.^41^ The diversity of the social sciences means that research is often produced across a range of research philosophies, which may not necessarily be complementary with one another. As a recent Perspective on this topic suggests, critical scholars that recognize that “evidence and knowledge are partial and unique to a given individual, context, and interpretation” are “unlikely to be comfortable sharing qualitative data with an eye toward synthesis and generalized analysis”.^85^^: 84^ Furthermore, research synthesis processes often have established expectations about how they operate and standards by which evidence is evaluated.^30^^,^^32^ Existing hierarchies of evidence often promote randomized control trials and quantitative data but can also limit the extent to which the insights from critical and interpretive social science can be included.^86^^,^^87^

Mainstreaming the full diversity of social science scholarship into environmental research will require careful and concerted work. The critical and interpretive social sciences require particular attention. Inspired by recent efforts to establish “ten facts” for informing land system research^88^ and similar papers that seek to bring social science insights into environmental research,^89^ here, we set out ten facts from the critical and interpretive social sciences that capture key insights which we believe can inform continuing efforts to enhance the capacity of environmental knowledge to enable desired transformative change.

## Ten facts from critical and interpretive social sciences

Facts are collectively held truths about the world that help communities to think and work productively together by establishing a shared reality and ways of working.^90^ In science, facts are produced through the organized collection, sharing and evaluation of evidence about a given phenomenon, which allows a defined peer community to agree about its nature.^91^ While facts from research are generated through located empirical enquiry, they typically move toward becoming “common sense” as they are increasingly accepted within society.^92^ The facts set out here, which we understand to be important for the pursuit of positive environmental and social outcomes, have been identified based on the authors’ collective training and experience working in and on interdisciplinary environmental research programs. To identify and synthesize these facts, we held a series of three participatory workshops involving social and interdisciplinary scientists alongside the establishment of a ten-year interdisciplinary research program called the Leverhulme Center for Nature Recovery at the University of Oxford.

**Figure 4:**
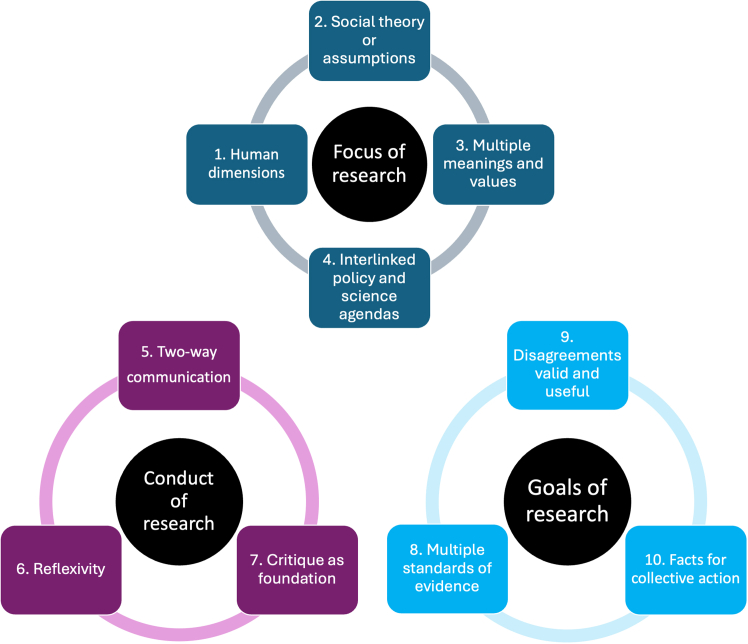
Working with the ten facts has implications for the focus, conduct, and goals of environmental research

The facts can be clustered according to their contribution to rethinking the focus, conduct, and goals of environmental research (Figure 4). However, the facts can also be used individually or in different arrangements as a resource for research teams and organizations to enhance capacity building and collaboration across disciplinary traditions.^93^ The ten facts do not speak for all critical and interpretive social scientists and they are not a panacea to overcoming noted barriers to interdisciplinary working.^94^^,^^95^ However, we hope that they can support a renewed agenda for alternative modes of doing research that help deliver on transformative ideals. A longer worked example of how each fact can be actioned as part of a larger research project follows.

### Fact 1. Human dimensions are ever present in environmental issues

The social aspects of environmental problems are sometimes termed their “human dimensions”.^38^^,^^96^ Human dimensions include the effects of culture and tradition, public and stakeholder engagement, and conflict management and resolution among others. In work on large-scale marine protected areas, for example, Christie et al. (2017) show how the world’s oceans which are often treated by oceanographic science as “unpeopled” are in fact shaped, studied, managed, used, and lived in by humans in a myriad of ways.^97^ Their work recognized human dimensions by widening the range of perspectives setting the research agenda for marine protected areas through participatory processes with marine protected area designers, managers, and researchers.^97^ Ensuring that human dimensions are acknowledged, even when not the main focus of a given research project, is deemed critical to doing justice to the lives and livelihoods of people in the environments where research is focused.^98^

### Fact 2. All environmental research involves social theory or assumptions

Critical and interpretive social scientists recognize that individual and collective assumptions shape environmental research and determine the insights of what that research makes visible and invisible.^99^^,^^100^^,^^101^^,^^102^ Good practice in social theory involves making explicit the mental models used by researchers to describe and explain the workings of the social world.^54^^,^^103^ Social theory can be used by researchers to predict and explain how and why things happen, i.e., why a given government might opt for market-based approaches to environmental governance over regulatory approaches. In research into Nature-based solutions to climate change and biodiversity loss, for example, Woroniecki et al. (2020) use social theories on knowledge and power to analyze how Nature-based solutions are produced, and identify sites of intervention to enhance promised social change and empower marginalized groups.^104^ Even research that does not focus on humans and their institutions (e.g., atmospheric physics or animal behavior) can include implicit social assumptions about the appropriate conduct of research, including choices about what to focus on and how.^47^ This means that any claims of neutrality (or detached objectivity) in research are difficult to maintain.^105^ Instead, being explicit in research outputs about the mental models and assumptions adopted can lead to more transparent, effective and equitable evaluation of findings and associated policy options.^41^^,^^106^^: 50^

### Fact 3. Multiple meanings and values matter in society

Interpretive research recognizes that understandings of reality are shaped by language, culture and history.^41^ Critical and interpretive social scientists are trained to identify and grapple with human tendencies to attach multiple meanings and values to entities, such as land^88^ or biodiversity^107^ (sometimes termed pluralism). In the book “Why We Disagree About Climate Change”, for example, Hulme reflects on the different meanings the climate holds for different social groups and argues that these culturally produced and value-laden meanings are both the reason for disagreements about climate change and the pathway to addressing the climate crisis.^108^ Although different communities look at environmental problems in different ways, which are sometimes contradictory to the initial views of scientists,^109^ careful research can document and understand these contextually defined meanings without leading to extreme relativism, where shared realities are non-existent.^41^

### Fact 4. The boundaries between research and policy are not always clear

Value-free science is a fallacy.^110^ Research that seeks to address issues of societal concern rarely maintains neat boundaries between the domains of science and policy.^111^ First, the focus of research itself is often influenced by political discourse and funding choices^112^^,^^113^; and, second, researchers have to make choices about how to conceptualize any issue, which can have political implications in terms of how the benefits of research and possible policy effects are distributed.^114^ As Turnhout et al. (2016) note: “the construction of policy relevant knowledge is a political act that involves choices about the preferred audiences of knowledge and the types of policy actions that may follow from this knowledge”.^74^^: 67^ Recognizing this, an analysis of environmental governance targets by McDermott et al. (2023), for example, concludes that embedding equity into the processes of environmental governance —including goal setting and the research that supports it— can better help to address the underlying social and economic challenges that are associated with environmental issues.^75^

### Fact 5. Two-way communication improves outcomes

Effective communication of environmental research can be as much about listening to and learning about what other groups of people already think, as it is about telling people what science reveals about an issue.^115^^,^^116^^,^^117^ In an article by Stefanoudis et al. (2017), for example, the authors offer insights about how to navigate and overcome the historical and present issue of “parachute science” in marine research by adopting two-way communicative practices, including: collaborative research with local partners; joint agenda setting; leveraging and acknowledging local knowledges and skillsets; sharing literature beyond paywalls; and learning about local regulatory landscapes.^118^ There is a wealth of evidence-informed advice in the literature about knowledge co-production,^29^^,^^31^^,^^79^ participatory action research,^119^^,^^120^ and other non-extractive research approaches.^121^^,^^122^

### Fact 6. Reflexivity can help navigate tensions

In recent years, reflexivity as a research practice has gained increased recognition in environmental research communities.^123^^,^^124^^,^^125^^,^^126^ Reflexivity has been informed by feminist and decolonial research^101^^,^^127^^,^^128^ and can take the form of “self-critical sympathetic introspection and the self-conscious analytical scrutiny of the self as researcher”.^129^^: 82^ In research on flood management in the UK, for example, Whatmore and Landstrom (2002) describe how reflexivity was organized into meetings between scientists and community members, which created opportunities for both parties to jointly interrogate and revise the science behind flood management measures.^46^ In this way, reflexive research can strengthen the social contract between science and society by creating openings to listen to, learn from, and respond to what research participants and users are saying,^130^ especially about perceived benefits and harm from research.^131^^,^^132^

### Fact 7. Critique is a foundation of good research

Critical thinking enables the theory testing and application that supports fact-finding missions of normal science (i.e., research design, data collection, and analysis).^133^ It also underpins the propensity of the critical and interpretive social sciences to raise second-order questions about the functions and implications of science and policy themselves, including who will be impacted by them and who is empowered to make decisions.^55^ Taking critique as a constructive input into environmental research can help make research outcomes more appropriate, legitimate, and ethical.^134^^,^^135^ In Diaz et al.’s (2018) report on the work of IPBES, for example, they note critiques raised within the global expert body by experts from a wide range of disciplines and knowledges, including those of Indigenous peoples and local communities.^136^ Specifically, criticism arose of ecosystem services as an analytical framework, which prompted the development of an alternative analytical framework (Nature’s Contributions to People) that was deemed to be “more legitimate and, therefore, more likely to be incorporated into policy and practice”.^136^^: 270^ In this way, critique can also support productive research systems through facilitating institutional learning.^134^^,^^137^^,^^138^

### Fact 8. Standards of evidence are not the same everywhere

Each scientific field has different expectations about the most appropriate methods to produce authoritative knowledge for environmental decision-making.^113^ For example, while randomized control trials that tease apart explanatory variables are hailed as a “gold standard” in some domains,^87^ other fields of research favor “feminist objectivity” that requires the background and values of the researcher to be explained as part of the research methodology.^105^ As demonstrated, for example, by research on community forestry in Nepal in Nightingale (2003), different types of evidence (e.g., ecological oral histories and aerial photographs) can be productively gathered alongside each other “on their own terms” to explore and compare different ways of looking at a problem.^139^^: 83^ If the goal of much environmental research is to inform non-scientific actors so as to catalyze action, then it is important to also recognize that the standards of evidence held by different actors are likely to differ from those within science itself.^140^ This may mean, for example, that quantity does not equal quality of data; rather, there is a need to understand and work with the expectations of the social groups that are intended to use those data in the wider world.^141^^,^^142^ In the context of transformative change, the value judgements that are made about which empirical evidence —be it qualitative or quantitative— is important and which is not need to be revised to accommodate more diverse forms of knowledge.^40^

### Fact 9. Scientific disagreement can be valid and useful

Collective agreement by scientists —also known as scientific consensus— can be seen as both a hallmark of good science and crucial for political action.^143^ However, focusing on scientific consensus has been recognized as limiting society’s potential to tackle urgent issues such as climate change and biodiversity loss.^30^^,^^84^^,^^144^^,^^145^ Overemphasizing consensus —where everyone agrees on the terms of enquiry and the interpretation of results— as a requirement of authoritative science runs the risk of covering up fundamental value conflicts that are important to explore through political debate, supported by more diverse scientific research.^113^^,^^146^^,^^147^ In an analysis of IPBES, for example, White and Lidskog (2023) note a tendency for this expert body to adopt strategies that smooth over disagreements between different kinds of knowledge rather than pro-actively create space to more explicitly acknowledge disagreement and diversity in environmental expertise.^148^ Shared understanding of disagreements may be a more productive way forward.

### Fact 10. Facts still matter for collective action

The facts that matter in society are defined by cultural context and are established by agreement of the authorities in a given time and place.^90^^,^^91^ An analysis of expertise within the European Union, for example, has shown that the establishment of shared expert organizations that could provide an evidence-base that was jointly produced and consistent across the member states was crucial for enabling collective action between sovereign states.^149^ Facts allow communities to work together in this way because they represent shared beliefs about the world, agreement on the necessary foundations of evidence to support those beliefs, and the articulation of collective ideals about what matters and why in a given society.^150^ However, making meaningful facts that can motivate environmental action requires paying careful attention to how facts are produced.^151^ Thinking carefully about the appropriate production of facts —including who produces knowledge, where, why, and how— need not undermine science, but rather strengthen capacities to challenge falsehoods and unsubstantiated claims in both scientific and political arenas (i.e., post-truth politics). A more explicit articulation of the extensive labor, methods, theories, and perspectives that go into producing facts and a clear-eyed assessment of the benefits and costs associated with them are considered to strengthen their capacity to mobilize action.^152^

## A worked example of the facts in action from the Oxford Leverhulme center for nature recovery

In this section, we reflect on how the facts presented in this perspective might operate in practice by tracing their ongoing implementation as part of an interdisciplinary project on equitable land use in the forest-agriculture mosaic landscapes of Ghana. This project is based in the Leverhulme Centre for Nature Recovery at the University of Oxford, which brings together scholars from different disciplines to understand, support and deliver nature recovery, defined as “the activity of helping life on Earth to thrive by repairing human relationships with the rest of the natural world”.^153^ The Centre has a ten-year work program and seeks to take account of complexity through concurrent research projects across six themes (listed in Box 2).

Box 2 **Research themes from the Leverhulme Centre for Nature Recovery**

(1) Ecology: Testing the effectiveness of different ecological approaches for nature recovery to support biodiversity and the delivery of ecosystem services such as climate change mitigation and adaptation.
(2) Scale and Technology: Tracking and evaluating nature recovery at both fine resolution and large spatial scales utilizing state-of-the-art remote sensing, big data, and deep machine learning techniques.
(3) Society: Encompassing the governance and socio-cultural dimensions of nature recovery.
(4) Finance: Scaling finance and investment for rapid nature recovery at a global scale.
(5) Human Health and Wellbeing: Exploring, understanding, and determining those aspects of nature which directly contribute to improvements in physical and mental health and wellbeing.
(6) Integration: Developing a novel analysis and decision platform to integrate nature recovery into land-use and infrastructure planning, and exploring scenarios that can deliver local, national and international commitments to nature, climate change, and sustainable development.

In this section, researchers from the Society theme (also authors on this paper) critically reflect on how the ten facts are being actioned in their current research on “climate smart” cocoa production in Ghana. The project explores the governance and financing of nature recovery in Ghanaian landscapes with a specific focus on supporting equity in relationships between communities, forests, food security and livelihoods.

### Focus of research

The research question of this project focuses specifically on equity; as such, the presence of questions regarding culture, values, and power in the research is widely accepted among the interdisciplinary project team. The involvement of researchers from the Society theme at the start of the project ensured that these human dimensions were integrated into the research design (Fact 1: Human dimensions). Critical and interpretive social science methodologies are being used to learn directly from farmers about how the security, or insecurity, of their land tenure influences the likelihood that they will seek to increase their uptake of nature recovery interventions. This approach requires the team to explicitly acknowledge the theories and assumptions about the social world that they are examining (Fact 2: Social theory or assumptions). In this case, the team also ensures that these theories and assumptions are open to revision as they learn about different ways of conceptualizing who farmers are and what they do, and the reasons why local actors may take up or neglect various nature recovery interventions. Likewise, there is ongoing negotiation around the multiple meanings held by different actors in the system, be they tied to climate-smart cocoa and carbon credits, or to farmer identity and land management practices (Fact 3: Multiple meanings and values). The team recognizes the normative ideals (i.e., related to climate mitigation, food security, etc.) that are embedded in the research agenda itself, and acknowledge that these align with broader political agendas around sustainable development that have origins dispersed far from the mosaic landscapes in which many of the research participants live and work (Fact 4: Interlinked policy and science agendas). The blurring of science and policy agendas reminds the team that they are doing more than simply gathering facts to be translated from science to policy; rather, they need to remain aware of, and be willing to listen to, alternative agendas for research and action that emerge on the ground which may be just as salient to their research participants.

### Conduct of research

Navigating tensions within the project currently benefits from trusted communication built upon long-term relationships between the research team from Oxford, and practitioners, farmers, and researchers in Ghana (Fact 5: Two-way communication). The team has needed to invest up-front in relationship building and co-design with communities in Ghana to achieve mutual understanding. This has been enabled by the critical social science researchers’ training and experience in conducting research with human participants, which requires taking time to think about one’s position and identity in relation to the research project, its goals, methods and partnerships (Fact 6: Reflexivity). While tension sometimes emerges between researchers in different themes (as there are distinct ethical guidelines amongst disciplines), the project team attempts to overcome this with communication and relationship building amongst team members. Researcher reflexivity has so far helped to maintain a critical stance at the front and center of the project by ensuring that the research identifies both the opportunities and the risks of nature recovery interventions — increasing the potential for more effective and equitable conservation and climate-related interventions in the mosaic landscapes of Ghana (Fact 7: Critique as a foundation). This was ultimately enabled by including critical and interpretive social scientists at the research design stage.

### Goals of research

**Figure 5:**
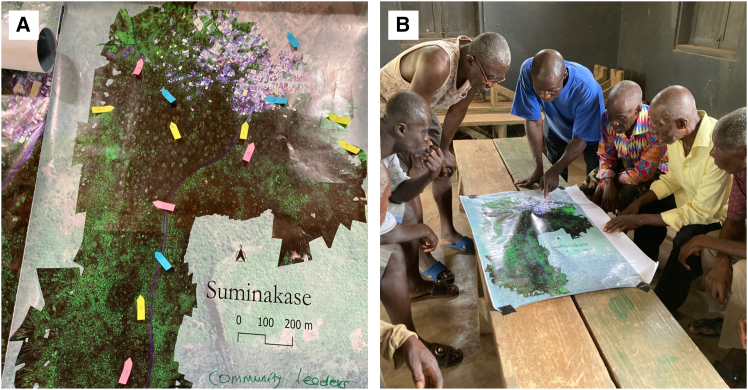
Two contrasting and complementary mapping approaches to understand the study landscape in Ghana (A) A high-resolution multispectral map of the study landscape in Ghana, produced using drone technology and ecological mapping methods by researchers in the Ecology, and Scale and Technology themes. (B) An image of a participatory mapping exercise with local community members to evaluate the map, run by researchers in the Society theme of the Leverhulme Center for Nature Recovery, Oxford. Photograph captured by Eric Mensah Kumeh with consent of all the participants for academic use.

Different groups of both knowledge producers and users (i.e., ecologists, social scientists, development practitioners and private companies) will hold different expectations of the kinds of knowledge needed to support socially just nature recovery in this context (Fact 8: Multiple standards of evidence). Differing standards of evidence have already presented some tensions between researchers; however, navigating these tensions has opened avenues for collaboration that in the long-run may produce better research. For example, researchers within the Scale and Technology and Ecology themes created a high-resolution aerial map of the study landscape (Figure 5A). However, as this did not fully meet the standards of evidence from social science, researchers from the Society theme ran gender-disaggregated participatory mapping exercises with the local community to evaluate the map (Figure 5B). These exercises revealed that one area, which the high-resolution aerial map initially showed as farmland, was in fact a landscape of rocks covered in grasses, thereby producing a more accurate map of that landscape and its features. The disagreements which may arise from different perspectives —be they between different disciplines or between scientists and farmers— on any problem were deemed valid and useful for understanding underlying value tensions about appropriate courses of action (Fact 9: Disagreements are valid and useful). Ultimately, the aim of the work is to develop research insights that can simultaneously help mitigate climate change and enhance the livelihoods of cocoa farmers (Fact 10: Facts help collective action). For this, research needs to generate shared understandings about the context in which climate-smart cocoa is being taken up and useful knowledge that will compel action.

As this worked example attests, the insights represented by the ten facts are starting to be actioned in ongoing projects in Ghana, as the team integrates insights from the critical and interpretive social sciences to adapt to environmental, societal, and political changes and provide a better understanding of the dynamics of climate change. However, there is more to do to foster a critical and interpretive ethos across the entire project team. As Nightingale et al. (2019) note, building “ontological plurality” in environmental research is difficult but crucial to illuminate knowledge gaps and pose new and important questions across disciplines.^154^ Open communication among members of the team, where the focus, conduct, and goals of research can be discussed and institutional learning can take place, is seen as an important enabler for this continuing effort.

## Outlook

Looking to the future, the contribution of these facts to effective and equitable environmental research necessitates the active inclusion and leadership of theory, insights, and researchers from the critical and interpretive social sciences. To support change, the ten facts need to be actively taken up, shared, and applied by research leaders at the outset of new interdisciplinary work. Some proposed actions for different kinds of researchers are set out in Table 1. These facts are not an endpoint in this conversation, but rather a provocation for continued exploration for how the critical and interpretive social sciences can be better enabled in environmental research.

**Table 1:** Proposed actions to harness the potential of these ten facts from the critical and interpretive social sciences

All researchers	Critical and interpretive social scientists	Other researchers	Organisations
• Read the facts as a conversation starter, rather than as a list of rules. • Curate epistemic agility by forging relationships and dialogue with researchers from other disciplines. • Recognise one’s own hierarchies of knowledge and be open to these being challenged and revised, where desirable. • Be open and humble to the limits of one’s knowledge and the limited scope of any given research.	• Find other scientists with whom to discuss questions about the ideas in this paper. • Contribute constructively by generating and sharing wider lessons from critical and interpretive research, while acknowledging its limitations. • Work with trusted scientists and other science communicators to share critical insights with wider audiences. • Where appropriate, acknowledge the need for pragmatic responses to urgent environmental problems and work with others to identify imperfect but important solutions.	• Find a critical and interpretive social scientist with whom to discuss questions about the ideas in this paper. • Seek out training in social theory and methodologies before carrying out social science research, especially when involving human participants. • Draw on the expertise of others and learn to recognise and work with disagreement and ambiguity in how things are interpreted and valued.	• Ensure capacity and resources that can support learning in interdisciplinary research initiatives are in place from the start. • Promote and fund environmental research initiatives that are co-led by and designed by critical and interpretive social scientists. • Approach these facts as educational in the short term, transformational in the long-term to support interdisciplinary research. • Champion research from all disciplines regardless of dominant attitudes or beliefs about hierarchies of knowledge.

For those who have not encountered the critical and interpretive social sciences in detail before, these ten facts are intended to provide a shareable resource on common critical and interpretive social science thinking that can support early career scientists through to research leaders who are seeking to engage in collaborative research programmes.^93^^,^^155^ Thinking like a critical and interpretive social scientist requires individuals to embrace and learn to navigate different research philosophies. This requires work. As such, the noted lack of university faculty teaching on environmental courses with foundational training in social theory^37^^,^^64^ poses a challenge to the natural growth of critical and interpretive scholarship in environmental research.

While encouraging discussion that fosters [interdisciplinary] learning (as proposed in Table 1) offers one leverage point for change, elevating the critical and interpretive social sciences in environmental research will also require overcoming entrenched mechanisms of marginalization. These mechanisms include, but are not limited to, the inequitable distribution of research funding and decision-making power — even in interdisciplinary spaces, which continue to be dominated by research leaders working with positivist research philosophies.^49^^,^^73^ The valuing of critical and interpretive social science research and their procedural inclusion in major environmental science initiatives will only be adequately enabled once power and resources have been equitably redistributed.^9^^,^^83^ Such redistribution may feel confrontational for environmental scientists and managers that are used to doing things a certain way. However, it is also a pathway to the transformative visions of a “below 1.5°” world and “living in harmony with nature” that governments and scientists around the world have committed to achieve. Marginalization in environmental science also reportedly plays out along “lines of gender, race/ethnicity, class, geography, and other dimensions of difference”.^73^ Elevating critical and interpretive social science to enable transformations will likely benefit from building solidarity and partnerships across movements and critical disciplines, such as research on degrowth, solidarity economics, Indigenous land management or agroecology, to “elevate and support alternatives that have the potential to supplant dominant cultural hegemony”.^49^^: 5^

There may also be continued reluctance from some critical and interpretive social scientists to contribute to mission-driven social and environmental policy agendas. They may, for example, be particularly conscious of possible social justice implications from these agendas that do not align with their values.^156^ However, environmental social scientists increasingly recognize the imperative to collaborate across disciplines and contribute by offering positive counter-visions that can improve or completely rethink these agendas.^46^^,^^53^^,^^157^^,^^158^^,^^159^ Toward this goal, the ten facts presented here do not replace the need for situated and careful analysis. They are intended to help promote the continuation of critical thinking, including about who wins, who loses and who decides across all facets of sustainability. They may, however, be useful in supporting early engagement with critical and interpretive social scientists in the framing and design of research programs. This could help keep assumptions open about the scope of the defined problem, while still working toward the development of solutions for sustainability challenges. They may also reinforce the case for critical and interpretive social scientists to be in leadership positions in initiatives such as global environmental assessments, and for them to be provided with the resources necessary to support involvement on their own terms.^83^^,^^160^ The facts also offer a reminder of the benefits of capturing diversity and disagreements in synthesis processes in order to attend more realistically to the wider world where human values are persistently in conflict.^8^

This article makes clear that social sciences which focus on environmental challenges are diverse.^21^ They bring philosophical and methodological pluralism to inform understandings of environmental problems, their impacts and appropriate solutions.^161^ Transformation toward more just and sustainable futures will require societies across scales, from the global to the local, to rework their relationships with each other and the world around them. We hope that foregrounding critical and interpretive social sciences in environmental research will lead to more robust, ethical and nuanced research on the environment, and support positive transformations toward just and sustainable futures for life on Earth.

## Acknowledgments

This work was supported by funding from the John Fell 10.13039/501100000769Oxford University Press (OUP) Research Fund, the 10.13039/501100000275Leverhulme Trust as part of its funding for the Leverhulme Centre for Nature Recovery of the University of Oxford, and the ANU Futures Scheme of the Australian National University. The authors would like to acknowledge valuable feedback from collaborators in the Leverhulme Center for Nature Recovery, anonymous reviewers and editors at One Earth in the development of this perspective.

## Author contributions

Conceptualization: J.M. and E.A.W.; funding acquisition: J.M. and E.A.W.; methodology: J.M. and E.A.W.; project administration: J.M. and E.A.W.; writing – original draft: J.M., E.A.W., L.M.A., Aoife Bennett, Andrea Byfuglien, S.B., H.F., B.G., C.H., M.H., E.M.K., V.M.-R., C.L.M., M.M., and L.P.; writing – review and editing: J.M., E.A.W., L.M.A., Aoife Bennett, Andrea Byfuglien, S.B., H.F., B.G., C.H., M.H., E.M.K., V.M.-R., C.M., M.M., and L.P.

## Declaration of interests

The authors declare no competing interests.

## Footnotes

Supplemental information can be found online at https://doi.org/10.1016/j.isci.2025.112736.
