# Supplementary material for: Ten facts from critical and interpretive social sciences for environmental research
Source: iScience. 2025 May 23;28(6):112736. doi: 10.1016/j.isci.2025.112736 (PMC12178800; doi:10.1016/j.isci.2025.112736)
Supplement: Document S1. Table S1 [file mmc1.pdf]

iScience, Volume *28*

Supplemental information

Ten facts from critical and interpretive social sciences for environmental research

Jasper Montana, E.A. Welden, Lea May Anderson, Aoife Bennett, Andrea Byfuglien, Sophie Bhalla, Hannah Fair, Beth Greenhough, Caitlin Hafferty, Mark Hirons, Eric Mensah Kumeh, Victoria Maguire-Rajpaul, Constance L. McDermott, Mari Mulyani, and Laura Picot

Table S1: Supporting data related to Figure 3.

Graph	Original source	Reference
1	"From 1990 to 2018, the natural and physical sciences received a total of USD 40 billion compared to only USD 4.6 billion for the social sciences and humanities (based on the means of the short and long search string results). In other words, according to our estimates, the natural and technical sciences received around 770% more funding than the social sciences and humanities for research on climate change."<br><br>Converted into percentage of total: 4.6 billion / 44.6 billion x 100 = 10.31% some social science content.	Overland, I., and Sovacool, B.K. (2020). The misallocation of climate research funding. Energy Research & Social Science *62*, 101349. https://doi.org/10.1016/j.erss.2019.101349.
2	Evaluation of all empirical research publications in Conservation Biology, Conservation Letters and Biological Conservation published in 2023. "Results across 533 papers showed that 32% of papers incorporated social science and that 64% of these social science papers investigated human action. Twenty-seven percent of these human action papers used explicit human action theories. The theory of planned behavior was the most used explicit theory (17% of action theory papers)."	Eyster, H. N., Gould, R. K., Chan, K. M. A., & Satterfield, T. (2025). Use of theories of human action in recent conservation research. *Conservation Biology*, *39*(2), e14461. https://doi.org/https://doi.org/10.1111/cobi.14461
3	Highest degrees identified for 35 members of the IPCC Executive Committee (10 members) and the IPBES Multidisciplinary Expert Panel (25 members). Of these, 26 held highest degrees in the biophysical sciences, 9 help highest degrees in interdisciplinary fields, specifically geography or environmental management and policy. None held highest degrees in traditional social science or humanities subjects.<br><br>Converted into percentage of total: 9/35 x 100 = 25.71% some social science content.	Unpublished data. Using publicly available profiles on IPCC and IPBES websites, or university/research profiles elsewhere online. Accessed 15/01/2025.
4 and 5	"Biological sciences subject areas were represented in 92% of the modules, whereas social sciences subject areas only featured in 60% and humanities in 24%. Of the modules teaching biological sciences subject areas, 84% included biological sciences faculty but only 31% of the modules covering social sciences subject areas included faculty from the social sciences."	Slater, H., J. Fisher, G. Holmes, C. Sandbrook and A. Keane (2024). "Assessing the breadth and multidisciplinarity of the conservation curriculum in the United Kingdom and Australia." BioScience: biae059. https://doi.org/10.1093/biosci/biae059
